# Does healthcare inequity reflect variations in peoples’ abilities to access healthcare? Results from a multi-jurisdictional interventional study in two high-income countries

**DOI:** 10.1186/s12939-020-01281-6

**Published:** 2020-09-25

**Authors:** Jeannie Haggerty, Jean-Frederic Levesque, Mark Harris, Catherine Scott, Simone Dahrouge, Virginia Lewis, Emilie Dionne, Nigel Stocks, Grant Russell

**Affiliations:** 1 St. Mary’s Research Centre and Department of Family Medicine, McGill University, Montreal, Quebec Canada; 2 Agency for Clinical Innovation and Centre for Primary Healthcare and Equity, University of NSW, Sydney, Australia; 3 Centre for Primary Healthcare and Equity, University of NSW, Sydney, Australia; 4 PolicyWise for Children & Families, Calgary, Canada; 5 Bruyère Research Institute, University of Ottawa, Ottawa, Canada; 6 Australian Institute for Primary Care and Ageing, La Trobe University, Melbourne, Australia; 7 St. Mary’s Research Centre, McGill University, Montreal, Canada; 8 Discipline of General Practice, University of Adelaide, Adelaide, Australia; 9 Department of General Practice, Faculty of Medicine Nursing and Health Sciences, Monash University, Melbourne, Australia

**Keywords:** Primary health care, Health services accessibility, Vulnerable population, Social characteristics

## Abstract

**Background:**

Primary healthcare services must respond to the healthcare-seeking needs of persons with a wide range of personal and social characteristics. In this study, examined whether socially vulnerable persons exhibit lower abilities to access healthcare. First, we examined how personal and social characteristics are associated with the abilities to access healthcare described in the patient-centered accessibility framework and with the likelihood of reporting problematic access. We then examined whether higher abilities to access healthcare are protective against problematic access. Finally, we explored whether social vulnerabilities predict problematic access after accounting for abilities to access healthcare.

**Methods:**

This is an exploratory analysis of pooled data collected in the Innovative Models Promoting Access-To-Care Transformation (IMPACT) study, a Canadian-Australian research program that aimed to improve access to primary healthcare for vulnerable populations. This specific analysis is based on 284 participants in four study regions who completed a baseline access survey. Hierarchical linear regression models were used to explore the effects of personal or social characteristics on the abilities to access care; logistic regression models, to determine the increased or decreased likelihood of problematic access.

**Results:**

The likelihood of problematic access varies by personal and social characteristics. Those reporting at least two social vulnerabilities are more likely to experience all indicators of problematic access except hospitalizations. Perceived financial status and accumulated vulnerabilities were also associated with lower abilities to access care. Higher scores on abilities to access healthcare are protective against most indicators of problematic access except hospitalizations. Logistic regression models showed that ability to access is more predictive of problematic access than social vulnerability.

**Conclusions:**

We showed that those at higher risk of social vulnerability are more likely to report problematic access and also have low scores on ability to seek, reach, pay, and engage with healthcare. Equity-oriented healthcare interventions should pay particular attention to enhancing people’s abilities to access care in addition to modifying organizational processes and structures that reinforce social systems of discrimination or exclusion.

## Background

**Fig. 1:**
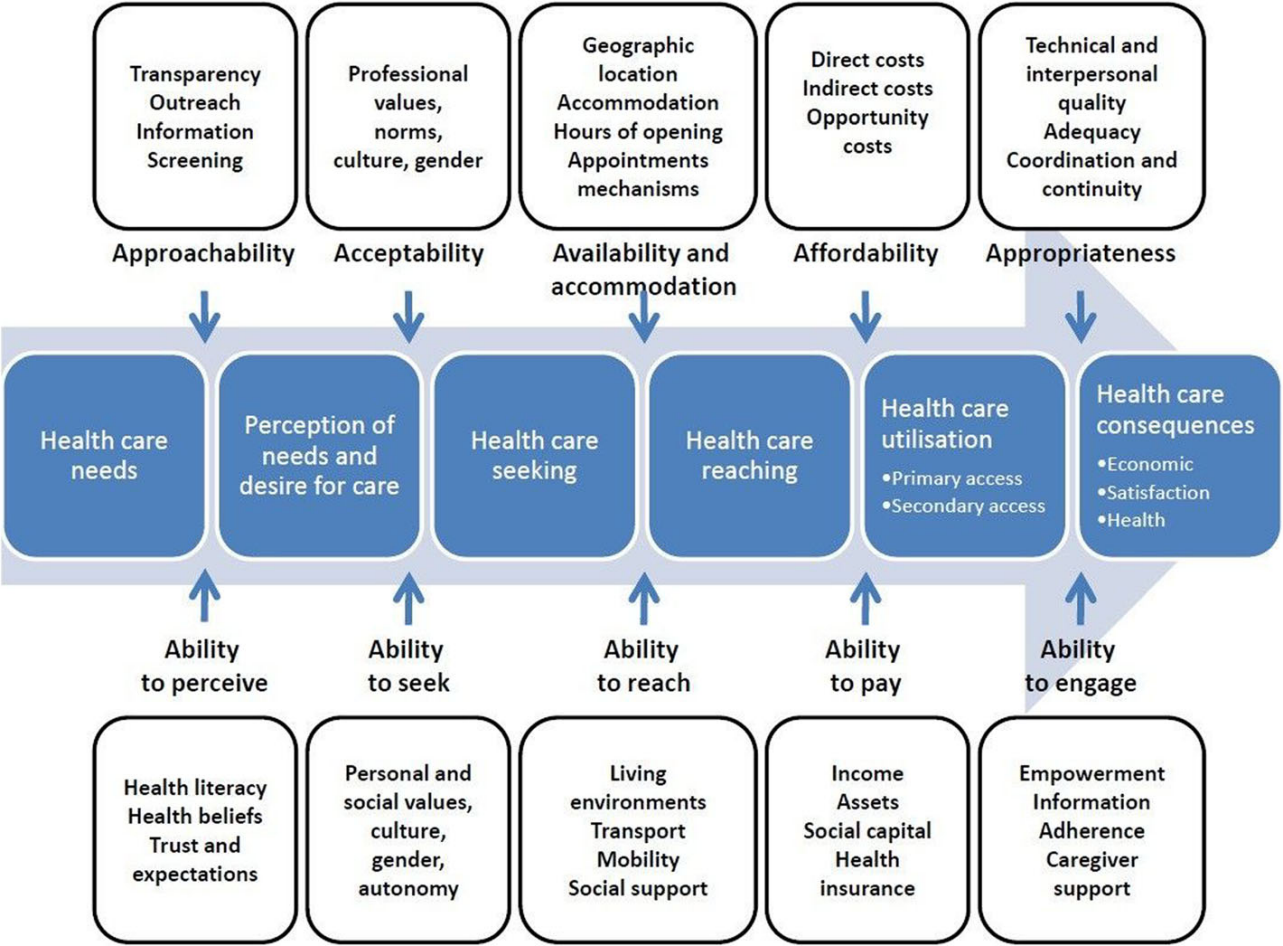
Patient-centered accessibility framework. Patient-centered access to healthcare framework depicting how five access abilities of the population interact with five dimensions of organizational accessibility to produce appropriate and effective healthcare [5]

Equitable access to primary healthcare is central to high performing healthcare systems [1] and is tied to better population health outcomes [2–4]. Primary healthcare services must be organized to offer a timely and appropriate response to the healthcare-seeking of diverse persons with a wide range of personal and social characteristics. The patient-centred accessibility framework (Fig. 1) [5], sees appropriate access to health care as being an interaction between people’s ability to perceive need, to seek appropriate options, to reach services, to afford direct and indirect costs, and to engage with service providers and five dimensions of organizational accessibility. Persons with excellent abilities to navigate the different obstacles of the care-seeking process may succeed in getting appropriate care for their needs, whereas those with low abilities will experience problematic access unless the organization has made specific efforts to make care approachable, acceptable, available & accommodating, affordable, and, ultimately, appropriate. Inequitable access occurs when access varies according to personal and social factors rather than according to need for care.

Increasing access to primary healthcare has been a health system priority in both Canada and Australia [6, 7] and has been the focus of an international participatory action-research program, IMPACT (Innovative Models Promoting Access-to-Care Transformation). This 5-year Canadian-Australian research program was built upon a network of local innovation partnerships that brought researchers in primary healthcare together with decision-makers, clinician leaders, and in some cases members of vulnerable communities in six regions (three in each country) with the aim of identifying, implementing, and trialling promising practice interventions to improve access to primary healthcare for vulnerable populations. Inspired by the patient-centred access framework (Fig. 1), the IMPACT innovations focused on redesigning organizational dimensions of the delivery of healthcare services.

This paper tests the assumption that socially vulnerable populations would exhibit lower abilities to access and would, therefore, be at higher risk of consequences of problematic access. Specifically, we define problematic access as reported difficulties in accessing care; forgone care; utilization of the emergency room (ER); and hospital admissions. Use of the hospital ER is frequently used as an indicator of problematic access to primary healthcare [8, 9] as are unplanned hospital admissions for ambulatory sensitive conditions [10].

The literature shows that persons who are at greater risk of social exclusion experience more difficulties with access to healthcare than their less socially vulnerable counterparts. The literature also shows that frequent ER use and higher hospitalization rates are associated with socioeconomic deprivatio006E, such as low income, low education, and immigrant or Indigenous status [11–16]. Socially vulnerable persons are less likely to receive appropriate care to manage their health problems [11, 17]. However, these studies do not shed light on whether difficulties in accessing care are due to differences in the abilities to perceive, seek, reach, pay for, and/or engage with healthcare or whether other social and organizational factors are at play.

In this article, we first examine how personal and social characteristics are associated with the abilities to access described in the Patient-Centered Access Framework and with the likelihood of reporting problematic access. We then examine whether higher abilities to access are protective against problematic access. Finally, we explore whether social vulnerabilities predict problematic access after accounting for abilities to access.

## Methods

### Design

This is an exploratory analysis of pooled data collected in the IMPACT study prior to the implementation of study interventions. The methods for the broader study have been previously described [18].

### Setting & participants

The IMPACT program was delivered in six regions (three in Canada; three in Australia) and targeted different populations, access abilities, and interventions (Supplemental Table 1). This paper’s analysis is based on the four study regions that had at least 25 participants who completed the pre-intervention survey data; the two sites with less than 25 participants at the time of analysis were excluded. The pooled sample is conceived as a sample of the target population of persons with multiple social vulnerabilities. All participants are adults (aged 18 years or older) who consented to participate in the evaluation and were able to respond to the questionnaires in one of the languages offered (English, French, Arabic).

### Data source

Data came from the pre-intervention survey that was administered in-person or by telephone to participants at the time of study enrolment. Data were collected between 2016 and 2018.

### Variables

Although the interventions differed, we used common questionnaire items across sites. We actively solicited information on 10 common chronic illnesses and asked about the presence of others in an open question. The sum of chronic illnesses (chronic illness burden) was used as a proxy of healthcare need.

#### Access ability measures

Because specific validated instruments have not been developed for the five abilities to access, we selected items from a variety of validated instruments that were judged to adequately map onto each construct. We subsequently used principal components analysis to reduce the number of variables into the relevant access dimensions (Supplemental Table 2) . Table 1 shows the operational definitions for the five abilities to access and how each was measured. Note that we do not have a measure of ability to perceive healthcare need. Note also that our measure of ability to pay does not measure ability per se but rather forgone care due to costs; therefore, the values are reversed compared to other abilities as this is a measure relevant to predominantly publicly-funded healthcare systems [19].

**Table 1:** Operational definition, measures, and score distributions for each access ability

Operational Definition Ability to…	Measure	Score Mean (SD)	Score Median (Q1; Q3)
**Perceive:** capacity to identify health need and the need to seek care	(no measure)	–	–
**Seek:** knowledge about available and acceptable healthcare options and right to receive care and personal autonomy to choose	Ease^1^ of finding health information by self, which services have right to receive, finding needed healthcare, deciding which health professional to see (4 items, range 1–4)	2.8 (0.8)	3.0 (2.3; 3.5)
**Reach:** capacity to overcome personal mobility and/or transportation barriers to travel to site of care	Ease of travelling to usual clinic (1 item, range 1–4)	3.4 (0.9)	4.0 (3.0; 4.0)
**[In]ability to Pay:** capacity to meet direct and indirect costs of care without negative impact on basic needs	Reported inability to pay for prescribed medications, laboratory tests (2 items, range 1–3)^2^	1.1 (0.3)	1.0 (1.0; 1.0) 16.7%, at least one barrier
**Engage:** ability to participate actively in decision-making and treatment decisions, including self-management	**Explain:** Ease of explaining needs to health professionals (1 item, range 1–4)	3.2 (1.0)	3.0 (2.0; 4.0)
	**Manage:** Does doctor or nurse give you sense of control over health, confidence to take care of health (2 items, range 1–4)^3^	3.3 (0.9)	3.5 (3.0; 4.0)

^1^ Response options: 1, not at all easy; 2, not very easy; 3, moderately easy; 4, very easy

^2^ How often did you NOT take 1) prescribed drugs or 2) laboratory tests because of cost: 1, never; 2, occasionally; 3, often

^3^ Response options: 1, not at all; 2, not really; 3, to some extent, 4, definitely

*Abbreviations*: *SD* standard deviation

To explore whether there exists a cumulative effect of abilities to access, we created a summary score of sum of abilities by dichotomizing each access ability at the median (below = 0; above = 1) then summing across abilities to access. The sum of abilities varies from 0 (below the median on all abilities to access) to 4 (above the median on all abilities to access) and includes ability to seek, ability to reach, and the two measures of ability to engage. We excluded ability to pay from the sum of abilities because it is conceptually and metrically different from the other abilities.

#### Personal & social characteristics

We captured patients’ socio-demographic profiles (see variables in Table 2 and Supplemental Table 3) using standard questionnaires. With the exception of age and sex, we categorized the social variables to represent meaningful categories of increasing social vulnerability, with the highest valued category representing the greatest social vulnerability based on our previous empirical results (Haggerty, manuscript in preparation) and the literature.

**Table 2:** Distribution of personal and social characteristics in the study sample

Personal and social characteristics	Overall *n* = 284 Percentage (n)
**Proxy of healthcare need**	
**Chronic illness burden**, % (n)	
Number self-reported chronic illnesses:	
0	17.3% (49)
1–2	25.4% (72)
3–5	28.5% (81)
6+	28.9% (82)
**Demographic & personal characteristics**	
**Mean age**, y (SD)	54.4 (16.3)
**Female**, % (n)	63.0% (179)
**Immigrant status**, % (n)	
Native born	70.7% (188)
Old immigrants	25.6% (68)
New immigrants (< 10 years)	3.8% (10)
**Language spoken at home**, % (n)	
Dominant language^1^	89.9% (249)
Other language only	10.1% (28)
**Indigenous or Aboriginal**, % (n)	6.7% (19)
**Social vulnerability characteristics** (in order of increasing vulnerability)	
**Self-perceived financial status**, % (n)	
Comfortable	32.5% (88)
Moderate	42.4% (115)
Poor or very tight	25.1% (68)
**Highest education level**, % (n)	
Post-secondary	61.1% (154)
High school	24.6% (62)
Less than high school	14.3% (36)
**Risk of social isolation**^2^, % (n) Persons for social support:	
Low (5–6 persons)	64.7% (161)
Medium (3–4 persons)	18.9% (47)
High (0–2 persons)	16.5% (41)
**Sum of social vulnerabilities**	
**Sum of social vulnerabilities**	
Sum of social vulnerability indicators: Indigenous or Aboriginal, new immigrant, financially poor, low education level, high risk of social isolation	
Mean (SD)	0.65 (0.78)
Median, 25th, 75th	0, 0
Percent with sum of 2+	12.7% (34)

^1^ Presumed proficiency with the dominant language (English, or French in Quebec; speaking only non-dominant language at home)

^2^ Social support was derived from number of persons available (0, 1, or 2+) for assistance with tasks of daily living, love and affection, confidante, good times

*Abbreviations*: *SD* standard deviation

Again, to explore the cumulative effect of social vulnerability, we dichotomized the social variables so that the category with the highest vulnerability was scored as 1 and other categories as 0. The sum of social vulnerabilities varies theoretically between 0 (no high vulnerability) and 6 (Indigenous or Aboriginal, new immigrant, limited proficiency in dominant language [English, or French in Quebec], financially poor, low educational level, and high risk of social isolation).

#### Problematic access

All access and utilization measures were self-reported. We elicited whether or not, in the previous 6 months, the person had experienced difficulties accessing primary healthcare, forgone care due to access issues, presented to the ER (both due to access difficulties and for any reason), or been admitted to hospital.

### Analysis

To examine the relationship of personal and social characteristics to both abilities to access and problematic access, we generated separate hierarchical logistic regression models to account for data being clustered within each region (level 2). We used a random intercepts model with independent variables entered as fixed effects. The minimum sample of 25 participants per site allows minimally reliable comparisons between regions in a multi-level model; we did not establish an a priori overall sample size requirement. Because of our small sample size (*n* = 284), we used a two-tailed alpha of *p* < 0.10 to indicate possible associations in the exploratory stages and the traditional critical value of *p* < 0.05 in the regression models.

Hierarchical linear regression models were used to explore the effects of personal or social characteristics on the different access indicators. We used separate models for each dichotomous indicator of problematic access, controlling for chronic illness burden as a proxy of healthcare need and exploring the effects of both abilities to access and personal and social characteristics. For the final model exploring the joint effect of abilities to access and personal and social characteristics, we used the sum of abilities to access and the sum of social vulnerabilities.

The odds ratio (OR) was derived by exponentiating the coefficient for the relevant independent variable in the logistic regression model. It represents the increase or decrease in the odds of the outcome associated with either a unit increase in a continuous variable (abilities to access, sum of abilities, sum of social vulnerabilities) or in the most vulnerable category of a social characteristics compared to the others assessed as non-vulnerable. We categorized independent variables to meet the formal assumption of linearity of the logit function for logistic regression modelling. We did the initial analyses using Generalized Estimating Equations in SAS software, Version 9.4 (SAS Institute Inc., Cary, NC, USA) with fewer formal assumptions, but we report the results obtained by Generalized Linear Modelling for mixed methods in SPSS Statistics for Windows, Version 24.0 (IBM Corp., Armonk, NY, USA). The results were consistent across both methods.

## Results

The distribution of the abilities to access measured in the study sample are shown in the last columns of Table 1. The values were skewed toward higher abilities; only a minority of subjects reported low abilities to access. There were significant differences between sites in abilities to access (see supplemental tables) as well as personal and social characteristics, justifying the need to account for site in the analysis.

The first column of Table 2 shows the characteristics of the study sample. The variables display the categorization used for analysis, with ordinal values increasing with increasing vulnerability. Despite a high proportion of immigrants overall (29.3%), only 3.8% had been in the country for less than 10 years (new immigrants). Only 10.1% spoke only a language different from the dominant language of the setting at home (predominantly Arabic in NSW). Alberta (AB) had a young and Indigenous and immigrant population with very low social support; Ontario (ON), a middle-aged, mainly female primary care practice population with low income and significant chronic illness burden; Quebec (QC), a younger relatively healthy population; and New South Wales (NSW), an older, chronically ill immigrant population. The most notable between-site differences were in financial status (ON poorest), Indigenous/Aboriginal status (AB highest) and use at home of non-dominant language (NSW highest; Arabic). As expected, these differences in personal and social characteristics reflect the type of population targeted by the intervention in each site (see supplemental tables).

Table 3 shows the association between personal and social characteristics and the different abilities to access healthcare. Only statistically significant or suggestive results are shown; there was no association by sex or level of education. The β coefficient is the statistically significant average increase or decrease in the ability score for each unit increase in the category of each personal or social characteristic. Neither chronic illness burden nor increased age are associated with lower scores in abilities to access, but characteristics typically associated with increased social vulnerability are associated with lower scores in abilities to access. For illustration, compared to those who perceive their financial status as “comfortable,” those who identify as “moderate” have on average 0.37 lower scores on ability to seek (β = − 0.37), and those reporting “poor or very tight”, 0.74 lower (2 X β = − 0.74). When the indicators of highest social vulnerability are summed, every unit increase in sum of social vulnerabilities is associated with lower scores in ability to seek (β = − 0.23) ability to engage (β = − 0.27); and the sum of abilities (β = − 0.41). The strongest and most consistent relationships are seen for self-perceived financial status and sum of vulnerabilities.

**Table 3:** Association of ability to access care with social vulnerability^1^

Personal and social characteristics (Ordered by increasing vulnerability, first category = 0)	Access ability					Sum of abilities β (95% CI)
	Seek β (95% CI)	Reach β (95% CI)	(Inability) Pay β (95% CI)	Engage-explain β (95% CI)	Engage-manage β (95% CI)	
**Proxy for healthcare need**						
**Chronic illness burden** Number self-reported chronic illnesses:	**–**	**–**	**–**		**–**	**–**
0				**- 0.17 (− 0.31; − 0.03)**		
1–2						
3–5						
6+						
**Demographics and personal Characteristics**						
**Increasing age, in** decades	**–**	**–**	**−0.05 (− 0.08; − 0.02)**	**–**	**0.14 (0.07; 0.21)**	**0.11 (0.02–0.21)**
**Immigrant status**	**–**			**–**	**–**	**–**
Native born		−0.24 (− 0.50; 0.02) *p* = 0.07	−0.09 (− 0.18; 0.01) *p* = 0.09			
Old immigrants						
New immigrants (< 10 years)						
**Language spoken at home**	−0.39 (− 0.79; 0.01)	**–**	**–**	**–**	**–**	**–**
Dominant language^2^	*p* = 0.06					
Other only						
**Indigenous or Aboriginal**	**–**	**–**	**–**	**–**	**–**	**–**
**Social vulnerability characteristics**						
**Self-perceived financial status**		**–**				
Comfortable	**− 0.37 (− 0.60; − 0.13)**		**0.12 (0.02; 0.22)**	**− 0.53 (− 0.80;** **− 0.27)**	**− 0.49** **(− 0.74;** **− 0.25)**	**− 0.78 (− 1.11;** **− 0.46)**
Moderate						
Poor or very tight						
**Risk of social isolation** Persons for social support:	−0.24 (− 0.52; 0.03) *p* = 0.09	**–**	**–**	**− 0.35 (− 0.68; − 0.02)**	**−0.36 (− 0.67; − 0.05)**	−0.35 (− 0.75; 0.05) *p* = 0.09
Low (5–6 persons)						
Medium (3–4 persons)						
High (0–2 persons)						
**Sum of social vulnerabilities** (score range 0 to 4)	**−0.23 (−0.36; − 0.10)**	**–**	0.05 (− 0.001; 0.11) *p* = 0.06	**−0.27 (− 0.42; − 0.12)**	**−0.27 (− 0.42; − 0.12)**	**−0.41 (− 0.59; − 0.23)**
**Most socially vulnerable** (0 or 1 vs. 2+ vulnerabilities)	**−0.35 (− 0.65; − 0.05)**	**–**	**–**	−0.30 (− 0.65; 0.06) *p* = 0.10	**−0.42 (− 0.76; − 0.08)**	**−0.67 (− 1.10; − 0.25)**

^**1**^ Average increase or decrease in each ability to access care associated with a unit increase in social vulnerability of personal and social characteristic. All effects are univariate in separate hierarchical linear regression models with the ability as the outcome variable and the personal or social variable as the independent variable. The first category is value = 0; other categories are valued at 1 or 2. β coefficients are shown only if significantly different from zero at *p* < 0.05. *p* values shown if 0.10 < *p* < 0.05

^2^ Dominant language was English, or French in Quebec

*Abbreviations*: *CI* confidence interval, *SD* standard deviation

The association between financial vulnerability and inability to pay appears modest (β = 0.12) because only 16.7% of the study population experienced at least one episode of forgone care due to cost. However, we found that those who self-identify as poor are 2.6 times more likely forgo prescribed medication or recommended tests because of cost compared to their finanacially moderate or comfortable counterparts.

Among the 284 participants, 88.3% (*n* = 233) indicated having needed health services or medical advice in the previous 6 months. Of these, 37.8% (88/233) reported difficulties accessing care (half more than once) and two-thirds (55/88) reported that these difficulties resulted in forgone care (higher in Canada than Australia). Over a quarter, 28.1% (80/284), reported ER use, of which 40% (32/80) reported using the ER because of difficulties accessing needed healthcare (higher in Canada than in Australia, *p* ≤ 0. 001). Twelve percent (12.3%) reported being hospitalized in the previous 6 months (Table 4).

**Table 4:** Indicators of access difficulties

Characteristic	Indicator of access difficulty (outcome variable in regression model)				
	Difficulty getting needed care or advice^**1**^ (*n* = 233)	Forgone care due to difficulty (*n* = 231)	Use of ER due to difficulty (*n* = 273)	Any ER use (*n* = 279)	Any hospitalization (*n* = 269)
**Frequency overall**	31.0%	19.5%	11.3%	28.2%	12.3%
**Chronic illness burden**	–	–			
Category of increasing burden			**1.73 (1.14; 2.62)**	**1.48 (1.12; 1.96)**	**1.49 (1.03; 2.15)**
**Impact of personal and social characteristics** OR (95% CI)					
**Increasing age,** decades	**0.71 (0.57; 0.87)**	**0.66 (0.25; 0.84)**	**0.67 (0.51; 0.89)**	**0.80 (0.66; 0.97)**	–
**Immigrant status** ref. = no; 1 = yes	–	–	–	**2.14**^**2**^ **(1.14; 4.00)**	–
**Limited language proficiency** ref. = dominant language^3^ at home, 1 = other language only	–	3.58 (0.83; 15.45) *p* = 0.09	–	–	–
**Indigenous/Aboriginal status** ref. = no; 1 = yes	–	–	–	**3.13 (1.14; 8.57**)	–
**Financial vulnerability** ref. = modest or comfortable, 1 = poor or very tight	**2.54 (1.32; 4.87)**	**3.36 (1.61; 7.00)**	**3.30 (1.45; 7.51)**	**2.08 (1.15; 3.78)**	–
**Sum of social vulnerabilities,** in order of increasing vulnerability	–	**1.59 (1.05; 2.40)**	**2.08 (1.30; 3.34)**	**2.09 (1.46; 2.97)**	–
**2+ Social vulnerabilities** ref. = less than 1, 1 = 2 or more	2.19 (0.97; 4.91) *p* = 0.06	**2.86 (1.19; 6.85)**	**3.18 (1.24; 8.19)**	**3.33 (1.57; 7.08)**	–

^1^ Among persons needing any healthcare in last 6 months

^2^ New immigrants show statistically higher any ER use: OR = 8.89 (95% CI: 2.09; 37.8)

^3^ Dominant language was English, or French in Quebec

Abbreviations: CI, confidence interval; ER, emergency room; OR, odds ratio

The likelihood of problematic access varied by personal and social characteristics, as shown in Table 4. The different problematic access outcomes are shown in the columns. Each OR is from a separate logistic regression model and denotes the increased/decreased likelihood of the problematic access outcome for the most vulnerable category compared to the rest. Again, only statistically significant or suggestive results are shown; there was no association by sex, level of education or risk of social isolation. In general, problematic access decreases by approximately 20% with every decade of increasing age but increases with increased social vulnerability. For instance, compared to those who report their financial status as “moderate” or “comfortable”, those reporting “poor” are 2.54 times more likely to report difficulties accessing care in the last 6 months and are over three times more likely to have forgone care (OR = 3.36) or to have used the ER (OR = 3.30) as a result of the difficulty. Those reporting at least two social vulnerabilities were more likely to experience all indicators of problematic access except hospitalizations. Only chronic illness burden was associated with hospitalization.

In contrast, Table 5 shows the effect of abilities to access on the likelihood of problematic access where higher access ability is protective against most of the indicators of problematic access except hospitalizations (Table 4). As an example, every unit increase in ability to seek care decreases the likelihood of experiencing difficulty getting needed care or advice by one-third (OR = 0.36). When the regression model is used to calculate probabilities of problematic access, those with the lowest score of ability to seek had a 0.90 probability of reporting difficulties getting needed care or advice compared to a probability of 0.35 for those with the highest score. Note that the direction of the effect is the opposite for (in)ability to pay. According to the model, those who indicate “occasionally” not getting medication and tests due to cost are 5.41 times more likely to report difficulties getting needed care or advice than those reporting “never” having this experience. Since inability to pay is associated with self-perceived financial status, the coherence in the directions and magnitudes of the OR for these variables in Tables 3 and 4 is not surprising.

**Table 5:** Impact of access abilities on indicators of problematic access^1^

Access Ability	Access difficulty in last 6 months (outcome in regression model)				
	Difficulty getting needed care or advice OR (95% CI)	Forgone care due to reported difficulty OR (95% CI)	Use of ER due to reported difficulty OR (95% CI)	Any ER use OR (95% CI)	Any hospitalization OR (95% CI)
**Ability to seek**	**0.36 (0.24; 0.53)**	**0.42 (0.28; 0.65)**	**0.60 (0.37; 0.97)**	**0.69 (0.49; 0.97)**	0.82 (0.53; 1.28) n.s.
**Ability to reach**	**0.62 (0.44; 0.90)**	**0.56 (0.38; 0.83)**	0.70 (0.45; 1.09) n.s.	0.75 (0.54; 1.05) *p* = 0.10	0.84 (0.55; 1.29) n.s.
**[In]ability to pay**	**5.41 (2.07; 14.2)**	**4.77 (1.91; 11.89)**	**3.71 (1.59; 8.69)**	**2.17 (1.01; 4.65)**	1.77 (0.73; 4.29) n.s.
**Ability to explain (Engage-1)**	**0.60 (0.44; 0.82)**	**0.65 (0.46; 0.91)**	**0.65 (0.44; 0.96)**	0.78 (0.59; 1.04) *p* = 0.09	0.91 (0.62;1.32) n.s.
**Ability to self-manage (Engage-2)**	**0.36 (0.24; 0.56)**	**0.40 (0.25; 0.62)**	**0.44 (0.28; 0.69)**	**0.60 (0.42; 0.84)**	0.93 (0.60; 1.44) n.s.
**Sum of abilities**	**0.41 (0.30; 0.55)**	**0.41 (0.29; 0.57)**	**0.58 (0.41; 0.82)**	**0.77 (0.61; 0.97)**	0.99 (0.73; 1.33) n.s.

^1^ OR indicates the increased likelihood of reporting the access difficulty associated with each unit increase in the access ability in separate logistic regression models, controlling for chronic illness burden as proxy of healthcare need

*Abbreviations*: *ER* emergency room, *n.s*. not significant, *OR* odds ratio

Although we did not find consistently a statistically significant association with hospitalization in these model, further analyses showed a statistically significant non-linear effect for financial status, educational status, social support, and Sum-of-vulnerabilities, as well as for ability-to-seek and ability-to-reach. Characteristically, likelihood curve for hospitalization is indeed highest for categories of highest vulnerability or lowest scores of ability to access care; it is lowest in the mid categories, and rises slightly again for the least vulnerable or with high levels of abilities to access care.

Finally, we explored the independent effects of abilities to access and social vulnerability on indicators of problematic access when adjusted for each other. For statistical efficiency and conceptual clarity, Table 6 shows the results of logistic regression models that included both sum of abilities and sum of social vulnerabilities. For each indicator of problematic access, there is some attenuation of both abilities to access and social vulnerability when controlled for each other, and only the sum of access abilities remains statistically significant for most indicators of problematic access.

**Table 6:** Likelihood of experiencing problematic access^1^

	Difficulty getting needed care OR (95% CI)	Forgone care due to difficulty OR (95% CI)	Use of ER due to difficulty OR (95% CI)	Any ER use OR (95% CI)
**Sum of access abilities**	**0.41 (0.30; 0.55)**	**0.46 (0.32; 0.65)**	**0.71 (0.49; 1.03)**	0.90 (0.70; 1.15) n.s
**Sum of social vulnerabilities**	0.89 (0.58; 1.34) n.s	1.21 (0.76; 1.92) n.s	**1.77 (1.07; 2.94)**	**1.92 (1.32; 2.78)**
**Chronic illness burden**	1.50 (0.99; 2.27) *p* = 0.054	**1.79 (1.09; 2.95)**	**2.15 (1.22; 3.77)**	**1.52 (1.05; 2.18)**
**Age,** in decades	**0.76 (0.61; 0.96)**	**0.73 (0.56; 0.94)**	**0.71 (0.53; 0.95)**	0.85 (0.69; 1.04) n.s

^1^ Results of multivariable logistic regression models with both sum of access abilities and sum of social vulnerabilities, controlling for age and chronic illness burden

*Abbreviations*: *CI* confidence interval, *ER* emergency room, *OR* odds ratio

## Discussion

In this sample of subpopulations selected in four different regions of two high-income countries according to their likelihood of experiencing difficulties with access to appropriate healthcare, we confirm findings from other studies that those at higher risk of social vulnerability are more likely to report problematic access (having difficulties getting needed care or advice, forgoing care and using the ER because of these difficulties, and higher use of the ER overall). What this study adds to the literature is that these social vulnerabilities are also associated independently with reported lower levels of ability to seek, ability to reach, ability to pay and ability to engage with healthcare. This confirms our previously untested assumption. Having two or more indicators of high social vulnerability significantly increases the likelihood of low ability to access healthcare and of problematic access. However, in regression models predicting problematic access, the aggregated measure of abilities to access remains independently and significantly protective against problematic access when adjusted for social vulnerability, and the independent effect of social vulnerability is greatly attenuated.

Our finding that socially vulnerable patients are more likely to experience problematic access to healthcare echoes what has been reported in the literature [14, 20–22]. In this small sample we found that effects are particularly marked for those who are financially poor and have two or more vulnerabilities. Although we only measured problematic access, a similar pattern is observed in other measures of appropriate care, such as recommended procedures for a variety of health conditions, preventive care, and mental health services, even in our universal healthcare systems [15, 23]. Although we did not find the same degree of disparity between native-born and immigrants that are reported in other studies [13, 15, 24], we observed higher ER use, especially among recent immigrants (< 10 years). Both Australia and Canada have publicly-funded health systems universally available to their citizens, but both have gaps that affect disproportionately the poor, new immigrants and other socially vulnerable populations [25–27].

Beyond the effect of specific indicators of social vulnerability, our findings support the notion of intersectionality in which multiple risk factors overlap and interact in a person to create a new state of amplified vulnerability, especially to effects that result from structures of privilege and power [28, 29]. We observe lower abilities to access and problematic access among those having two or more vulnerabilities, regardless of what the specific vulnerabilities are. An independent review [30] also found reports of greater access disparity associated with multiple vulnerabilities. Consequently, in our study, factors that were not individually predictive, such as low educational level and immigrant status, became predictive of problematic access in combination. It is critical for publicly-funded healthcare systems to reflect on how to ensure access for vulnerable groups, such as those living in poverty, those with severe mental health problems, and those experiencing other characteristics that make individuals susceptible to social exclusion. A lens of intersectionality calls for profound reflection on structures and processes in the health system that reinforce discrimination and social exclusion.

Investing in educational *and* organizational interventions to improve or enhance abilities to access in the population may be a promising approach to reducing disparities in access. Our finding that abilities to access are independently more predictive of problematic access than social vulnerability is particularly interesting and important because modifying abilities to access is more readily actionable than modifying social vulnerability. In the IMPACT interventions, we found a statistically significant improvement in participants’ ability to seek (from 2.9 to 3.2, paired t-test = 6.43, *p* > 0.001) and in their ability to explain their problems to health professionals (from 3.2 to 3.4, paired t-test = 3.73, *p* > 0.001). This occurred despite the fact that these were modest, low-cost interventions, such as providing navigation advice to access services and incentivizing the use of a diabetes self-management support website (also available in Arabic) through nurse-led health checkups. It needs to be highlighted that the interventions were initially intended as modifications of supply-side service delivery rather than directly targeting the enhanced abilities of the population. Moving forward, the results of the IMPACT research program suggest that interventions combining supply side re-design and demand side ability development are particularly promising to ensure access to healthcare for vulnerable people.

We acknowledge several limitations. First, the exploration of the effect of abilities to access on problematic access was a secondary objective of this research program, and despite the sophisticated statistical models used, the results remain exploratory in nature. We did not apply a correction to the critical value for statistical significance despite multiple testing because of the small sample size, but our conclusions focus more on the consistency of trends rather than estimates of effect, per se. Second, the measures of abilities to access are crude and not designed for this particular purpose. Their skewed distribution may result in less-than-optimal model specification. However, with skewed measures there is higher discriminatory capacity and information yield in the tail, which is the focus of our analyses. We anticipate that more precise and accurate measures would show a stronger rather than a weaker effect. Third, our small sample size limited our statistical power to demonstrate effects at α = 0.05 despite observing more frequent problematic access among Indigenous/Aboriginal, immigrants, and persons with low educational levels. Low numbers of these groups are likely due to challenges in responding to our survey instrument, despite efforts to facilitate the task of responding. Finally, our last finding showing that the sum of abilities seems to be more important than the sum of social vulnerabilities in predicting problematic access warrants particular attention. The effect of the sum of social vulnerabilities may be attenuated when adjusted for the sum of abilities because social vulnerability is on the causal pathway between abilities to access and problematic access. Such a finding could be explored by path models and structural equation modelling in a larger and independent sample. The fact that cumulative social vulnerabilities remain significant for some ER use, even when abilities to access are accounted for in the model, suggests that we need to continue to better understand how social characteristics produce a poor fit between demand and supply dimensions of accessibility.

## Conclusions

Despite these limitations, this exploratory study provides first evidence, in a small and varied sample, that some of the observed effects of problematic access in socially vulnerable populations are due to limited abilities: to seek, to reach, to pay for, and to engage with care. We also found that abilities to access were more predictive of problematic access than social vulnerability, which is encouraging because abilities to access are more modifiable than social and personal characteristics. This suggests that, when designing equity-oriented healthcare interventions, attention should be paid to actions that can enhance people’s abilities to access care in addition to modifying supply-side organizational processes and structures that reinforce social systems of discrimination or exclusion. This will increase first-contact access overall but is likely to more specifically benefit people who are socially vulnerable.

## Supplementary information

**Additional file 1: Table S1.** Summary of targeted populations, access issues, and interventions in the IMPACT program. **Table S2.** Principal components factor resolution for items measuring abilities to access healthcare. **Table S3.** Distribution of personal and social characteristics of each study sample and tests of significant differences between the four sites. **Table S4.** Distribution of scores for each measure of access ability (range 1–4, except as noted) for each study sample and tests of significant differences between the four sites.

## Data Availability

The datasets analysed during the current study are available from the corresponding author on reasonable request.

## Abbreviations

AB: Alberta
ER: Emergency room
IMPACT: Innovative Models Promoting Access to Care Transformation
NSW: New South Wales
ON: Ontario
OR: Odds ratio
QC: Quebec
