# Supplementary material for: Does healthcare inequity reflect variations in peoples’ abilities to access healthcare? Results from a multi-jurisdictional interventional study in two high-income countries
Source: Int J Equity Health. 2020 Sep 25;19:167. doi: 10.1186/s12939-020-01281-6 (PMC7517796; doi:10.1186/s12939-020-01281-6)
Supplement: Supplementary file 1 — Additional file 1: Table S1. Summary of targeted populations, access issues, and interventions in the IMPACT program. Table S2. Principal components factor resolution for items measuring abilities to access healthcare. Table S3. Distribution of personal and social characteristics of each study sample and tests of significant differences between the four sites. Table S4. Distribution of scores for each measure of access ability (range 1–4, except as noted) for each study sample and tests of significant differences between the four sites. [file 12939_2020_1281_MOESM1_ESM.docx]

**Supplemental Table 1. Summary of targeted populations, access issues, and interventions in the IMPACT program**

|  | **CANADA** | | | **AUSTRALIA** | | |
| --- | --- | --- | --- | --- | --- | --- |
|  | **ALBERTA** | **QUEBEC** | **ONTARIO** | **VICTORIA** | **NSW** | **SOUTH AUSTRALIA** |
| **Vulnerable group** | Immigrant, low income, Aboriginal, senior, and homeless populations | High deprivation neighbourhoods | Socially vulnerable patients | Low socioeconomic status, social isolation, chronic illness/disability | Low socioeconomic locality, culturally and linguistically diverse communities (including refugees) | Residential Aged Care Facility residents with complex medical needs |
| **Access issue** | Rural population with limited access to primary healthcare | Patients newly assigned to a family physician through a centralized wait list | Family practice patients needing access to community resources | Vulnerable clients of chronic disease or dental services with no family physician | Patients with poorly controlled diabetes attending family practices | Residential Aged Care Facility clients needing after hours care |
| **Intervention** | Pop-up primary healthcare services delivered at existing community events for hard to reach populations | Telephone outreach to help newly assigned patients prepare for their first visit with a family physician | A lay patient navigator supports primary care patients to reach community resources | A health broker links patients having no family physician to volunteer family practices | Health visits and a diabetes self-management website provide information and referrals | Facilities redesign procedures to improve afterhours care |

Abbreviations: IMPACT, Innovative Models Promoting Access-to-Care Transformation; NSW, New South Wales.

**Supplemental Table 2. Principal components factor resolution for items measuring abilities to access healthcare**

| **Items** | **Component^1^** | | |
| --- | --- | --- | --- |
|  | **1** | **2** | **3** |
| **Factor 1: Ability to seek** |  |  |  |
| How easy is it for you to find the healthcare you need? | **0.891** |  |  |
| How easy is it for you to find out which health services you have the right to receive? | **0.842** |  |  |
| In general, how easy is it for you to get health information by yourself when you need it? | 0.789 |  |  |
| How easy is it for you to decide which health professionals you need to see? | 0.739 |  |  |
| **Factor 2: Ability to engage** |  |  |  |
| Did this doctor or nurse give you a sense of control over your health? |  | 0.945 |  |
| Did this doctor or nurse help you feel confident about your ability to take care of your health? |  | 0.922 |  |
| **Factor 3: Cost barriers** |  |  |  |
| How often did you NOT take laboratory tests or exams that were prescribed by a doctor or nurse because of their cost (like blood draw, X-rays, etc.)? |  |  | 0.905 |
| How often did you NOT take drugs that were prescribed by a doctor or nurse because of their cost? |  |  | 0.728 |

^1^Factor scores are the average of individual question values and can be interpreted relative to the original Likert scales, where 1 = very easy and 4 = not at all easy.

**Supplemental Table 3.** **Distribution of** p**ersonal and social characteristics of each study sample and tests of significant differences between the four sites**

|  | **Overall**  n = 284 | **AB**  n = 63 | **ON**  n = 79 | | **QC**  n = 60 | | **NSW**  n = 82 | **Difference between sites** |
| --- | --- | --- | --- | --- | --- | --- | --- | --- |
|  | **Proxy of healthcare need** | | | | | | |  |
| **Chronic illness burden,**  mean number self-reported chronic illness (SD) | 3.8 (3.2) | 2.3 (4.0) | 3.7 (2.3) | | 2.0 (2.0) | | 6.2 (2.3) | F = 35.56,  p ≤ 0.0001 |
| **Chronic illness burden,** % (n) Number of self-reported chronic illnesses:  0  1–2  3–5  6+ | 17.3% (49)  25.4% (72)  28.5% (81)  28.9% (82) | 47.6% (30)  27.0% (17)  7.9% (5)  17.5% (11) | 8.9% (7)  22.8% (18)  48.1% (38)  20.3% (16) | | 20.0% (12)  50.0% (30)  21.7% (13)  8.3% (5) | | 0% (0)  8.5% (7)  30.5% (25)  60.1% (50) | Χ^2^ = 139.09,  df = 9,  p < 0.001 |
|  | **Demographic & personal characteristics** | | | | | | |  |
| **Mean age**, y (SD) | 54.4 (16.3) | 48.8 (18.4) | 53.51(18.8) | | 49.3 (13.9) | | 62.5 (9.2) | F = 12.21,  p < 0.01 |
| **Female**, % (n) | 63.0% (179) | 65.1% (41) | 75.9% (60) | | 61.7% (37) | | 50.0% (41) | Χ^2^ = 11.79, df = 3,  p = 0.01 |
| **Immigrant**, % (n)  n=266  Native born  Old immigrants  New immigrants (< 10 years) | 70.7% (188)  25.6% (68)  3.8% (10) | 93.5% (43)  6.5% (3)  0% (0) | 80.8% (63)  17.9% (16)  1.3% (1) | | 75.0% (45)  20.0% (12)  5.0% (3) | | 45.1% (37)  47.6% (39)  7.3% (6) | X^2^ = 42.79,  df = 6,  p < 0.001 |
| **Indigenous or Aboriginal,** % (n)  n=284 | 6.7% (19) | 20.6% (13) | 3.8% (3) | | 1.7% (1) | | 2.4% (2) | Χ^2^ = 25.48, df = 3,  p ≤ 0.0001 |
|  | **Social characteristics,** in order of increasing vulnerability | | | | | | |  |
| **Self-perceived financial status**, % (n)  n=271  Comfortable  Moderate  Poor or very tight | 32.5% (88)  42.4% (115)  25.1% (68) | 25.5% (13)  49.0% (25)  25.5% (13) | 16.5% (13)  46.8% (37)  36.7% (29) | | 55.9% (33)  32.2% (19)  11.9% (7) | | 35.4% (29)  41.5% (34)  23.2% (19) | Χ^2^= 28.06  df = 6  p < 0.001 |
| **Highest education level**, % (n)  n=252  Post-secondary  High school  Less than high school | 61.1% (154)  24.6% (62)  14.3% (36) | 61.3% (19)  25.8% (8)  12.9% (4) | 63.3% (50)  31.6% (25)  5.1% (4) | | 68.3% (41)  15.0% (9)  16.7% (10) | | 53.7% (44)  24.4% (20)  22.0% (18) | X^2^ = 13.54  df = 6  p = 0.035 |
| **Language spoken at home**, % (n)  n=277  Dominant language^1^  Other language only | 89.9% (249)  10.1% (28) | 86.0% (49)  14.0% (8) | 100% (79)  0% (0) | | 96.7% (53)  3.3% (2) | | 77.8% (63)  22.2% (18) | Χ^2^ = 25.96  df = 3  p ≤ 0.0001 |
| **Risk of social isolation**, % (n)  Persons for social support:  n = 249  Low (5−6 persons)  Medium (3−4 persons)  High (0−2 persons) | 64.7% (161)  18.9% (47)  16.5% (41) | 40.0% (12)  16.7% (5)  43.3% (13) | 65.4% (51)  21.8% (17)  12.8% (10) | | 63.3% (37)  23.3% (14)  13.3% (8) | | 74.1% (60)  13.6% (11)  12.3% (10) | X^2^ = 21.19  df = 6  p = 0.002 |
| **Sum of vulnerabilities** | | | | | | | | |
| **Sum of social vulnerability,** mean (SD)  Indicators: Aboriginal, new immigrant, financial status, education level, social support | 0.65 (0.78) | 0.93 (0.85) | 0.59 (0.67) | | 0.48 (0.72) | | 0.67 (0.83) | F = 3.21  p = 0.02 |
| **2+ indicators of high social vulnerability**, % (n)  n=267 | 12.7% (34) | 19.6% (9) | 10.1% (8) | 10.0% (6) | | 13.4% (11) | | X^2^ = 2.85  df = 3  p = 0.42 |

^1^ Dominant language was English, or French in Quebec.

Abbreviations: AB, Alberta; df, degrees of freedom; NSW, New South Wales; ON, Ontario; QC, Quebec; SD, standard deviation.

**Supplemental Table 4. Distribution of scores for each measure of access ability (range 1−4, except as noted) for each study sample and tests of significant differences between the four sites**

|  | **Overall**  n = 284 | **AB**  n = 63 | **ON**  n = 79 | **QC**  n = 60 | **NSW**  n = 82 | **Difference between sites** |
| --- | --- | --- | --- | --- | --- | --- |
| **Ability to seek**  Mean (SD)  Median  (Q1; Q3) | 2.8 (0.8)  3.0  (2.3; 3.5) | 2.6 (0.8)  2.5  (2.0; 3.0) | 2,7 (0.8)  2.8  (2.0; 3.3) | 2.7 (0.8)  2.8  (2.0; 3.3) | 3.0 (0.8)  3.3  (2.5; 3.8) | F = 3.72  p = 0.01 |
| **Ability to reach**  Mean (SD)  Median  (Q1; Q3) | 3.4 (0.9)  4.0  (3.0; 4.0) | 3.1 (1.3)  4.0  (1.5; 4.0) | 3.3 (0.8)  3.0  (4.0; 4.0) | 3.5 (1.0)  4.0  (3.0; 4.0) | 3.6 (0.8)  4.0  (4.0; 4.0) | F = 3.17  p = 0.03 |
| **Ability to pay (range 0−3, reverse order)**  Mean (SD)  Median  (Q1; Q3) | 1.1 (0.3)  1.0  (1.0; 1.0) | 1.3 (0.4)  1.0  (1.0; 1.0) | 1.2 (0.4)  1.0  (1.0; 1.0) | 1.1 (0.3)  1.0  (1.0; 1.0) | 1.1 (0.3)  1.0  (1.0; 1.0) | F = 4.08  p = 0.01 |
| **Ability to explain**  **(Engage1)**  Mean (SD)  Median  (Q1; Q3) | 3.2 (1.0)  3.0  (2.0; 4.0) | 2.6 (1.1)  2.5  (2.0; 3.5) | 3.0 (1.1)  3.0  (2.0; 4.0) | 3.4 (0.8)  4.0  (3.0; 4.0) | 3.4 (0.8)  4.0  (3.0; 4.0) | F = 6.38  p < 0.001 |
| **Ability to manage (Engage2)**  Mean (SD)  Median  (Q1; Q3) | 3.3 (0.9)  3.5  (3.0; 4.0) | 2.8 (1.1)  3.0  (2.0; 3.5) | 3.3 (0.7)  3.5  (3.0 ; 4.0) | 2.7 (1.0)  2.8  (1.8; 3.5) | 3.6 (0.8)  4.0  (3.5 ; 4.0) | F = 10.61  p < 0.001 |
| **Sum of abilities (range 0−4)**  Mean (SD)  Median  (Q1; Q3) | 2.3 (1.3)  2.0  (1.0; 3.0) | 1.6 (1.4)  1.0  (0.0; 3.0) | 2.2 (1.2)  2.0  (1.0; 3.0) | 1.8 (1.1)  2.0  (1.0; 3.0) | 3.0 (1.1)  3.0  (2.0; 4.0) | F = 17.36  p ≤ 0.0001 |

Abbreviations: AB, Alberta; NSW, New South Wales; ON, Ontario; QC, Quebec; SD, standard deviation.
